# Institutional Needs

**Published:** 1920-02-07

**Authors:** 


### Institutional Needs.

A CORRECTION.

IN commenting on the antiseptic properties and values of Iglodine (THE HOSPITAL, January 24, 1920, p. 397) the strength in which this substance acts as an efficient antiseptic and bactericide was stated to be *1 in 500*. This was an error for *1 in 50*, which is the correct strength. Those of our readers who are about to make a trial of Iglodine will please note that they must use it ten times as strong as our previous notice suggested.

